# Enhanced generation of retinal progenitor cells from human retinal pigment epithelial cells induced by amniotic fluid

**DOI:** 10.1186/1756-0500-5-182

**Published:** 2012-04-10

**Authors:** Fatemeh Sanie-Jahromi, Hamid Ahmadieh, Zahra-Soheila Soheili, Maliheh Davari, Shima Ghaderi, Mozhgan Rezaei Kanavi, Shahram Samiei, Abdolkhalegh Deezagi, Jalil Pakravesh, Abouzar Bagheri

**Affiliations:** 1 National Institute of Genetic Engineering and Biotechnology, Tehran, Iran; 2 Ophthalmic Research Center, Shahid Beheshti University of Medical Sciences, Tehran, Iran; 3 Iranian Blood Transfusion Organization Research Center, Tehran, Iran; 4 Department of Obstetrics and Gynecology, Aban General Hospital, Tehran, Iran

**Keywords:** Retinal progenitor cells, Amniotic fluid, Age related macular degeneration (AMD), Cellular therapy, Serum-free

## Abstract

**Background:**

Retinal progenitor cells are a convenient source of cell replacement therapy in retinal degenerative disorders. The purpose of this study was to evaluate the expression patterns of the homeobox genes *PAX6* and *CHX10* (retinal progenitor markers) during treatment of human retinal pigment epithelium (RPE) cells with amniotic fluid (AF), RPE cells harvested from neonatal cadaver globes were cultured in a mixture of DMEM and Ham's F12 supplemented with 10% FBS. At different passages, cells were trypsinized and co-cultured with 30% AF obtained from normal fetuses of 1416 weeks gestational age.

**Results:**

Compared to FBS-treated controls, AF-treated cultures exhibited special morphological changes in culture, including appearance of spheroid colonies, improved initial cell adhesion and ordered cell alignment. Cell proliferation assays indicated a remarkable increase in the proliferation rate of RPE cells cultivated in 30% AF-supplemented medium, compared with those grown in the absence of AF. Immunocytochemical analyses exhibited nuclear localization of retinal progenitor markers at a ratio of 33% and 27% for CHX10 and PAX6, respectively. This indicated a 3-fold increase in retinal progenitor markers in AF-treated cultures compared to FBS-treated controls. Real-time PCR data of retinal progenitor genes (*PAX6*, *CHX10* and *VSX-1*) confirmed these results and demonstrated AF's capacity for promoting retinal progenitor cell generation.

**Conclusion:**

Taken together, the results suggest that AF significantly promotes the rate of retinal progenitor cell generation, indicating that AF can be used as an enriched supplement for serum-free media used for the in vitro propagation of human progenitor cells.

## Background

With the current increasingly aging population, the incidence of age related macular degeneration (AMD) is expected to rise [1]. In recent years, AMD has been the main cause of irreversible vision loss in elderly individuals from industrialized nations [2,3].

Although a large volume of studies have been conducted to investigate palliative therapies and stop the progression of the disease, there is still no definite treatment for AMD [4].

A number of treatments have previously been used, some of which, in addition to not being suitable for retinal restoration, have been found to affect the adjacent healthy cells [5][8]. In parallel with the numerous attempts made to produce efficient medication, investigations by cell biologists have spurred novel curative strategies for retinal rehabilitation: "cell replacement therapy" [9][12].

The ability of stem cells to repair lost photoreceptors in the retina has opened a promising avenue to researchers [13][15]. In recent years, several sources of stem cells have been under investigation as a replacement for damaged photoreceptors. These include embryonic, marrow-derived and umbilical cord-derived stem cells, and immortalized cell lines [16][19]. However, of all stem cells, retinal derived progenitor cells may be a more efficient treatment for visual impairment [20]. More than 20 years have passed since the first report of retinal pigment epithelium (RPE) transplantation in animal models [21][23] and human trials [24]. These clinical studies have offered hope to ophthalmologists because of the competency of RPE cells in reviving previously disappearing cells^,^ net connection and visual function [18]. Despite the encouraging results, there are still difficulties associated with this kind of treatment, and more studies are required to overcome such obstacles. Amniotic fluid (AF) is enriched with a variety of growth factors and nutrients, and several reports have shown that it is necessary for embryonic cell proliferation, differentiation and dedifferentiation [25,26]. This study focused on how AF can lead to retinal progenitor cell development.

## Resuls

### RPE cell culture

**Figure 1:**
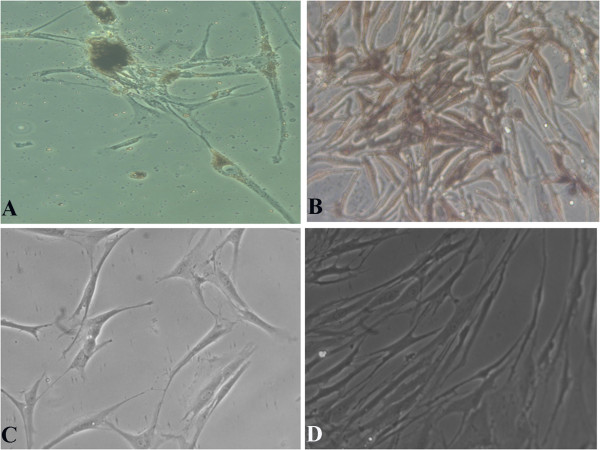
**Microscopic images of RPE cells.A**, **B**: Deeply pigmented RPE progeny, at the first passage, with different morphologies that had recently attached to the plate surface. **C**, **D**: Elongated RPE cells at late passages; demonstrating the appearance of peripheral processes and loss of pigmentation after several passages. Magnification: 200.

**Figure 2:**
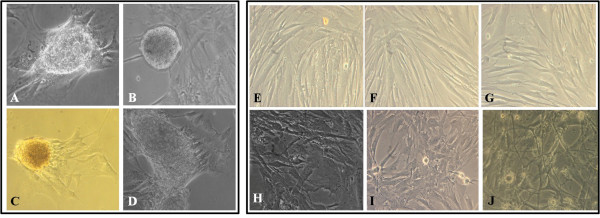
**Microscopic images of RPE cultures.A**: Expansion of spheroid colony from young cultures (passage 3). **B**: Free colony floating in supernatant (passage 3). **C**, **D**: Re-cultured free colonies and establishment of a new RPE monolayer (passage 5). **E**, **F**, **G**: Ordered arrangement of RPE cells cultured on AF-coated surfaces (passage 9) **H**, **I**, **J**: Chaotic spread of RPE cells grown on FBS-coated surfaces (passage 9). Magnifications: 200.

The enzymatic isolation of RPE cells from the globe, using dispase I, yielded a culture with a higher degree of RPE purity when compared with mechanical and other enzymatic methods of isolation. Small, pigmented RPE cells with differing morphologies adhered to the culture surface and proliferated (Figure 1A, 1B). Pigmentation decreased with increasing passage, culminating in the disappearance of all pigmented granules from around the nucleus. The cells also increased in size through repeated subcultures, and elongated peripheral processes emerged (Figure 1C, 1D). Nevertheless, some cultures exhibited spontaneously arising sizeable spheroid colonies that were detectable by eye (Figure 2A). These colonies, when allowed to continue their growth, evolved into unattached free colonies floating in the supernatant (Figure 2B). Trapping and reculturing the free-floating colonies led to the establishment of a new RPE cell monolayer. This confirms the potential of colonies to initiate another series of attachment and proliferation on the plate surface (Figure 2C-2D).

### Growth in AF

To further examine the cultures, trypsinized cells were gently centrifuged (5 min at 300*g*), the supernatants discarded and the residual precipitates were re-suspended in complete medium supplemented with 10% AF, 20% AF and 30% AF. Cells grown in AF-supplemented medium produced more established colonies than those grown in FBS, RPE cells cultivated in AF-coated flasks required only 15 min to adhere to the plate surface, compared to at least 90 min needed for FBS-coated flasks. In addition, cultures on AF-pre-coated surfaces displayed a clearly visible track of aligned RPE cells (Figure 2E-2G), while cells in FBS-supplemented medium did not show any regular spreading or specific positioning on the surface (Figure 2H-2J). Also the number of cells that attached to AF-coated surfaces was always greater than that of FBS-coated dishes (data not shown).

### Immunocytochemistry

**Figure 3:**
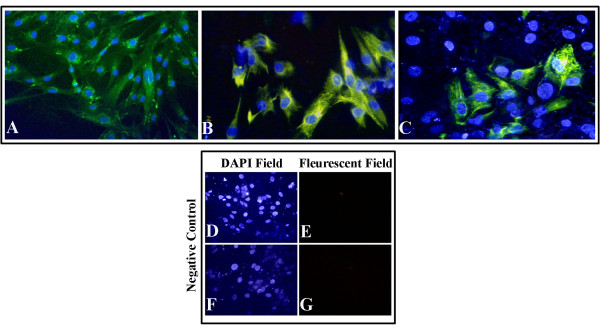
**Fluorescence microscopy of RPE cells.A**, **B**: Fluorescence microscopy of RPE cells from the first culture, indicating an RPE cell culture purity of nearly 100%. immunostaining procedure was carried out as described in the methods. **A**: Cytoplasmic and granular expression of RPE65 in cells positive for RPE65 after immunostaining with rabbit anti-human RPE65 polyclonal antibody, **B**: Cytoskeletal expression of cytokeratin 8/18 in RPE cells positive for cytokeratin 8/18, immunostained with mouse anti-human cytokeratin 8/18 antibody. **C**: Decrease in cytokeratin 8/18 expression at passage 7 of RPE cultures. **D**-**G**: Negative control for cytokeratin 8/18 (**E**) and RPE65 (**G**) expression, showing no non-specific binding of the aforesaid markers. As detailed in the methods section, the negative controls were not stained with any primary antibodies. Nuclei were counter-stained with DAPI. Magnification: 200.

Immunocytochemical data obtained from the first passage of RPE cells, freshly isolated from eye globes, indicated a high degree of purity for harvested RPE cells, with over 90% of the cells positive for RPE65 (Figure 3A) and cytokeratin 8/18 (Figure 3)B.

**Figure 4:**
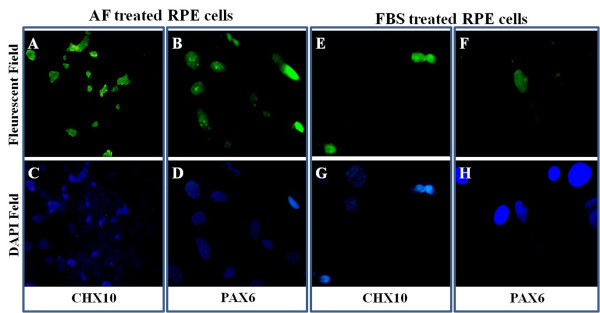
**Fluorescence microscopy of retinal progenitor marker expression in AF-treated (Left) and FBS-treated (Right) RPE cells.A**, **E**: Nuclear localization of CHX10 in cells (**A**: AF-treated cells, **E**: FBS-treated cells) immunostained with goat polyclonal anti-human CHX10 antibody. **B**, **F**: Nuclear localization of PAX6 in cells (**B**: AF-treated cells, **F**: FBS-treated cells) immunostained with goat polyclonal anti-human PAX6. **C**, **D**, **G**, **H**: The nuclei of each field were counter-stained with DAPI. Note that AF-treated RPE cultures have an approximately 3-fold increase in retinal progenitor marker expression (33% and 27% for CHX10 and PAX6, respectively) when compared with that of FBS-treated cultures. Each value represents the mean of 3 independent experiments in at least triplicate. Magnifications; **A** &**C**: 200, **B**, **D**, **E**, **F**, **G** &**H**: 400.

Rates of RPE65 and cytokeratin expression decreased with repeated subculturing, suggesting the appearance of undifferentiated retinal progenitor cells (Figure 3C). Cultures devoid of primary and/or secondary antibodies were negative for all examined markers in each case (Figure 3D-3G). Immunocytochemical analysis of RPE cells co-cultured with 30% AF showed 33% immunostaining for CHX10 (Figure 4A, 4C) and 27% for PAX6 (Figure 4B, 4D), which suggests an approximate 3-fold increase in expression of the aforesaid makers compared to FBS-supplemented medium (13% for CHX10 and 8.6% for PAX6) (Figure 4E-4H). In control DMEM/F12 cultures, immunocytochemical analysis showed a decrease in the number of cells positively expressing retinal progenitor markers (data not shown). CHX10 and PAX6 markers displayed a nuclear localization and they had a punctate distribution. Cultures devoid of primary and/or secondary antibodies were negative for all examined markers in each case (Figure 3D-3G).

### RPE cell proliferation and cell death ELISA assays

**Figure 5:**
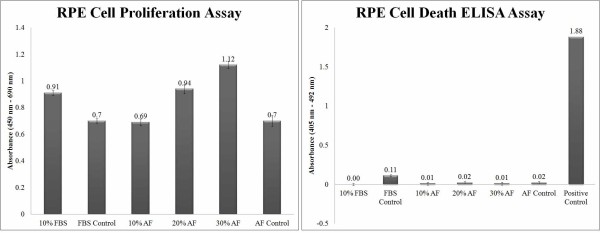
**Analysis of RPE cell proliferation (Left) and death (Right).** An ELISA assay was carried out as described in the methods. Left: Comparison of RPE cell proliferation between AF-treated cultures (10%, 20%, 30%), FBS-treated cultures (10%) and DMEM/F12 (control)-treated cultures. Note the dose-dependent increase in AF-treated RPE cell proliferation. Right: Comparison of RPE cell death in AF-treated cultures (10%, 20%, 30%), FBS-treated cultures (10%) and DMEM/F12 (control)-treated cultures with positive control (DNA-histone complex). Note that AF does not have any apoptotic effects on RPE cells at any of the concentrations tested. Comparative analysis of AF and FBS-treated cultures indicates that AF may be a suitable substrate for FBS replacement in culture medium. Each value represents the meanSEM of 4 independent experiments in at least triplicate.

RPE cell proliferation rates were evaluated in the presence of AF (10%, 20%, and 30%), FBS (10%), and DMEM/F12 as a control. The results suggest that AF-treated cultures, when compared with those lacking AF in the media, had a dose-dependent increase in proliferation, most likely due to the growth factor-rich content of AF (Figure 5 Left). Cell death analysis showed that there was no significant impact of AF on the apoptotic rate in RPE cultures which is comparable to that of FBS (Figure 5 Right).

### Real Time PCR

**Figure 6:**
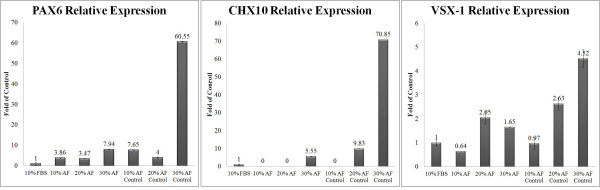
**Relative retinal progenitor gene expression in AF-treated cultures (10%, 20%, 30%), FBS-treated cultures (10%) and DMEM/F12 (control)-treated cultures.** RPE cell preparation and RNA extraction were performed as described in the methods. Relative gene expression was determined by quantitative real time PCR and normalized against GAPDH mRNA levels. The bar graphs show the normalized expression ratio in AF-treated RPE cells adjusted to FBS-treated cells. Left: Relative *PAX6* expression. Note the dose-dependent increase of *PAX6* expression in AF-treated RPE cultures and higher *PAX6* expression in AF-treated cultures compared with FBS-treated cultures. The slow up-regulation of *PAX6* expression may be evidence of an increase in the number of retinal progenitor cells. *PAX6* is up-regulated in AF (30%) control cultures, which may be an indicator that these cultures contain neuro-retinal terminally differentiated cells. Center: Relative *CHX10* expression Right: Relative *VSX-1* expression. Note the low level of *VSX-1* expression in comparison to expression of *CHX10*. Each value represents the meanSEM of 2 independent experiments in at least triplicate.

According to the RT-PCR data, *PAX6* expression levels increased when 3 dosages of AF (10%, 20%, 30%) were used to treat cultures, when compared to FBS cultures and that this elevation was highest when using 30% AF. Surprisingly, *PAX6* expression levels in DMEM/F12-treated cultures (control) increased several-fold compared to those of AF- and FBS-treated cells (Figure 6 Left).

*CHX10* expression was not detected when 10% and 20% concentrations of AF were used but was present in 30% AF-treated cells in which *CHX10* expression was significantly increased when compared to FBS-treated cultures (5.55 fold). Similar to *PAX6* expression, control cultures also displayed a much greater increase in *CHX10* expression levels (70.85 fold) (Figure 6 Center).

Although *VSX-1* expression levels increased with increasing doses of AF (10% and 20%), there was a slow decrease in expression levels in 30% AF*-*treated cultures. Overall, AF-treated cultures displayed higher *VSX-1* expression than FBS-treated cells. In the control cultures, the trend in level of *VSX-1* expression was similar to that of *PAX6* and *CHX10* (Figure 6 Right).

## Discussion

The results presented in this study show that AF is a robust promoter of growth for retinal progenitor cells. AF has an approximately neutral pH (7.2), and its osmotic pressure is in the physiological range, thus providing a suitable and appropriate environment for cell growth and proliferation. Previous studies have been carried out to identify the content of the AF proteome. Cho et al identified the 15 most abundant proteins in AF at gestational ages of 1618 weeks which included albumin, fibronectin, serotransferrin, complement C3, ceruloplasmin and TGF- [27].

Previous works have also suggested the ability of AF to initiate regeneration in damaged cells [25,26], thus confirming the data obtained in this study.

The innate capacity of adult somatic cells has many potential applications in regenerative medicine [28]. The retinal pigment epithelium begins as a plastic tissue, capable, in some species, of generating lens and retina but differentiates early in development and normally remains nonproliferative [29].

Our results show that RPE cells cultured on AF-coated surfaces displayed an organized alignment when compared to the disorganized spread on FBS-coated dishes. Although proteomic analysis of AF has not been the focus of this work, it seems that fibronectin, as the 3^rd^ most abundant protein in AF in the 16^th^ week of gestation [27], plays a pivotal role in making this organized alignment. Fibronectin is an extracellular matrix protein that has an essential role in cell attachment, polarity and migration [30].

The results also indicate that RPE cells grown in AF-containing medium require only 15 min for their initial attachment compared to at least 90 min required for those cultured in FBS-supplemented medium. Consistent with this observation, the report by Heth et al [31] demonstrates that cells grown on fibronectin- and laminin-coated microfilters required much less time to reach confluency when compared to collagen I-coated microfilters. Also, cell morphology was maintained better on fibronectin-coated microfilters, similar to our own observations in this study of AF-treated cells.

The increase in RPE cell proliferation and retinal progenitor gene expression levels in AF-supplemented medium were found to be dose-dependent. The cell proliferation ELISA, immunocytochemistry and RT-PCR data showed the ability of AF to induce retinal progenitor genes and thus convert an RPE culture into an invaluable source of retinal progenitor cells. It is likely that such a dose-dependent increase in proliferation and regeneration is due to the presence of growth factors whose concentration correlates with cell proliferation and regeneration. The effect on RPE cells of several of these growth factors, including TGF-, complement C3, albumin, plasminogen, ceruloplasmin and serotransferrin, has been examined previously. As suggested by Saika, following the formation of a wound in the tissue, the TGF- factor is activated, turning on a series of signaling pathways involved in proliferation and regeneration [32]. There are several other reports indicating the role of TGF- in epithelial mesenchymal transition (EMT), cell migration to the area of damage and the establishment of regeneration [33]. Complement C3 is another factor in AF that has been found to be responsible for the regeneration of damaged tissue. Kimura et al suggested that complement C3 plays a role in inducing cell proliferation and is specifically expressed in wounded lens tissue for tissue regeneration [34]. Reca et al also confirmed the presence of C3 receptors on hematopoetic stem cells directing the cells towards damaged tissue [35]. Plasminogen is also a factor involved in cell proliferation and wound healing. Ceruloplasmin, 1 microglobin, serotransferrin, apolipoprotein A and albumin are other AF proteins essential for cell homeostasis and transport.

In agreement with previous reports on the effect of separate growth factors on RPE cells, the results of this study suggest that AF is a valuable composite with all the aforementioned factors, and therefore represents a powerful supplemental medium.

Here, we have shown that AF was able to promote retinal progenitor gene expression levels in 30% AF control cultures, while immunocytochemical analysis of 30% AF control cultures indicated a decrease in the number of cells positive for the retinal progenitor markers. Furthermore, ELISA cell proliferation data showed a decreased rate of proliferation in 30% AF control cultures. Taken together, these results suggest that *PAX6* has a governing role and is a master regulatory gene located upstream of *CHX10* and *VSX-1*. Similar to the study of Hsieh et al, the *PAX6* level in proliferating progenitor cells is determined by the cell, and its level depends on the cell cycle phase. On the basis of Hsieh et al study, a low level of *PAX6* expression is crucial for cells to re-enter S phase of the cell cycle and therefore complete proliferation. Therefore, a very high level of *PAX6* expression represses further cell proliferation [36]. Our ELISA and real-time PCR results are consistent with this hypothesis. According to the Hsieh et al study, neural cells are able to express *PAX6* to 3 distinctive extents: low (confined to neural progenitor cells), high (confined to pre-neurogenic progenitors, differentiated neural cells, amacrine cells and retinal ganglion cells) and negative or zero (confined to cone photoreceptors and bipolar cells). Taking this into consideration, and considering that for each sample the same numbers of cells were examined, our results show that *PAX6* overexpression does not signify progenitor cell genesis. Our immunocytochemical and RT-PCR data derived from the 30% AF control cultures show similar results. The analyses of *PAX6* expression levels show that RPE cells treated with 30% AF contain the greatest number of retinal progenitor cells of the tested cultures. In addition, *CHX10* and *VSX-1* expression levels in 30% AF-treated cells indicate the presence of early and late retinal progenitor cells, respectively. *PAX6* overexpression in 30% AF control cultures shows that these cultures contain neural differentiated cells; *CHX10* and *VSX-1* expression levels in the control cultures suggest that neural cells could represent a range of differentiated and undifferentiated bipolar cells and/or Muller cells, although additional analysis of bipolar markers should be carried out to confirm this. In 10% AF- and 20% AF-treated cultures as well as 10% AF control cultures, *PAX6* was expressed at a low level, which may be an indicator of the presence of retinal progenitor cells. *VSX-1* expression in these cultures confirms the existence of late retinal progenitor cells; however, the lack of *CHX10* expression must be investigated further. According to a study by Dhomen et al, the absence of *CHX10* expression at a late stage during the progenitor cell cycle leads to the continuation of progenitor cell proliferation in the adult retina [37]. Therefore, further experiments are needed for these cultures.

The reason for the lack of *CHX10* expression in 10% AF, 20% AF and 10% AF control cultures is unclear. A 5-fold increase in *CHX10* expression in 30% AF-treated cultures suggests a slow expression of the *CHX10* gene in early retinal progenitor cells. Rapid up-regulation of *CHX10* expression in 30% AF control cultures suggests a quick rise in the number of bipolar and/or Muller cells in these cultures.

A significant finding of this study is that AF does not modify retinal progenitor gene expression patterns. This is very significant with regard to the treated cells that are to be accepted for use in future experiments. For example, *VSX-1* expression in all treated cultures was at a lower level than *CHX10* expression. This pattern has also been reported by several other studies [36]. This indicates that *CHX10* can negatively regulate *VSX-1* expression. AF-treated (10% and 20%) cultures were negative for *CHX10* expression while *VSX-1* showed a dose-dependent increase in expression levels. In the 30% AF-treated cultures, a further increase in *VSX-1* expression was expected, but surprisingly, *VSX-1* expression levels dropped, which may be due to the increase in *CHX10* expression in the 30% AF-treated cases and its subsequence negative regulation of *VSX-1* expression. According to several reports, *CHX10* mostly acts to repress its target genes, *VSX-1* is a *CHX10* target gene. In fact, Clark et al have demonstrated that a high expression of *CHX10* is always in accordance with a low expression of *VSX-1* and vice versa [38].

We also examined cultures for their ability to differentiate into other cellular components of the retinal layer; specific retinal cell markers (PKC and CRABPI, (unpublished data) Rod and Thy1.1 [39]) were examined in the presence of AF using immunocytochemistry and real-time PCR. These experiments confirmed that retinal progenitor cells are able to generate retinal terminally differentiated cells such as bipolar cells, amacrine cells, rod photoreceptors and retinal ganglion cells [39].

## Conclusion

Several studies have focused on the significance of cell replacement. Tissue engineering and cell replacement therapy are becoming more established therapeutic interventions as more experiments are performed in this area. Stem cells and their use in the treatment of retinal diseases offer an encouraging source for transplantation. However, the lack of sufficient access to relevant RPE cells and the current debate on the issue of stem cell applications have created obstacles for researchers with regard to acquiring appropriate suitable sources of cells for transplantation.

Retinal progenitor cells, if available, can offer the greatest opportunity, ability and potential for transplantation. This study shows that amniotic fluid has the potential to induce RPE cells to form retinal progenitor cells and therefore represent a readily available source of retinal progenitor cells for future retinal therapies.

## Methods

### Cell culture

Pathogen-free post-mortem human neonatal eye globes, with no previous ophthalmic disease, were obtained from the Central Eye Bank of Iran, RPE cell cultures were established under sterile conditions. Post-mortem procedures were carried out between 2448 h after death. Dissection and sampling of the globe was carried out as described below. Fat and other intruding peripheral tissues of the eye were removed using fine scissors. The vitreous was removed via a narrow split between the iris and sclera, and the vitreous and the interior of the globe was flushed with a strong stream of PBS to remove the remaining neural tissues. Several more intensive washes of PBS were used to eliminate blood and other adjacent tissue impurities, exposing the pigmented RPE layer to the washing buffer. The entire RPE layer was carefully detached from the underlying tissue and dissected into 2 mm^2^ pieces, which were then incubated in the presence of dispase I (1.1 U/ml) (Gibco, Germany) for 50 min at 37C. The loosened tissue, along with the released RPE cells, was consequently centrifuged (300*g* for 5 min, at 4C). The supernatants were discarded, and the resulting pellets were cultured in 25 cm^2^ flasks (Nunc. Denmark) containing a mixture of DMEM and Ham's F12 at a 1:1 (v/v) ratio (Sigma, Germany) supplemented with 20% FBS (Gibco), 50 g/ml of gentamycin (Darupakhsh. Co, IRAN), 120 g/ml of penicillin (Fluka, China), 220 g/ml of streptomycin (Fluka, China) and 250 g/ml of fungosine (Gibco). The flasks were then incubated in an incubator at 37C with a humidified atmosphere of 5% CO_2_. The culture medium was typically exchanged with 10% FBS-supplemented medium once a week until the cells were 80% confluent. Thereafter, subculturing was performed at a ratio of 110^6^ cells per 75 cm^2^ of flask surface. Fully confluent cultures from early (13), mid (47) and late (8, 9) passages were employed in the subsequent experiments.

### Amniotic fluid preparation

Amniotic fluid samples were obtained from thirty pregnant women who underwent amniocentesis for the assessment of genetic deficiencies in the first trimester of gestation. Amniotic fluid cells were removed for karyotype analysis. The remaining supernatants, in cases with no evidence of chromosomal abnormalities, were pooled and used in our downstream experimental procedures. The collection of these samples was approved by the ethics committees of the NIGEB and the Ophthalmic Research Center. The AF samples were centrifuged at 300*g* for 5 min at 4C, and the resulting supernatants were then sterilized using a 0.2 m membrane filter (OrangeScientific, Belgium) and stored at 70C until the time of analysis.

### Immunocytochemistry

RPE cells from early and mid passages (data shown are derived from passage 6), were cultured on FBS or AF pre-coated glass cover slips in a 24-well microplate (Nunc, Denmark) at a density of 110^5^ cells per well and incubated for 24 h. After incubation, the medium of each well was changed to either experimental (10% FBS and 30% AF-supplemented medium), or control (DMEM/F12 with no AF or FBS) medium (Table 1). Seven days after incubation, standard immunocytochemistry was performed according to the Santa Cruz protocol. RPE cells were fixed and permeabilized with pre-chilled methanol (10C) (Merck, Germany) for 5 min at room temperature and then blocked using 1% BSA (Merck) in PBST (1% Triton X-100 in PBS) (Sigma) for 20 min at room temperature. Antibodies for retinal progenitor cell markers included the goat polyclonal anti-human PAX6 and goat polyclonal anti-human CHX10. Rabbit anti-human polyclonal RPE65 was used as a specific RPE cell marker and mouse anti-human monoclonal cytokeratin 8/18 as an epithelial cell marker (All antibodies were obtained from Santa Cruz, USA). All primary antibodies were used at a dilution of 1:50 in 1.5% BSA in PBST and incubated for 1 h at room temperature. Cells were washed with PBS to avoid any non-specific background immunostaining. A negative secondary antibody-only control was also included. FITC conjugated antibodies (donkey anti-mouse for PAX6 and CHX10, goat anti-rabbit for RPE65 and goat anti-mouse for cytokeratin 8/18, Santa Cruz, USA) were used at a dilution of 1:100 in 1.5% BSA in PBST and incubated for 45 min at room temperature in the dark. A background control with neither primary nor secondary antibody was used for each marker. Finally, nuclei were counter-stained with DAPI (1 mg/ml, Santa Cruz, USA) to assess of the total number of cells in each field. Cover slips were then mounted onto slides using an anti-fading mounting medium (90% glycerol, 10% PBS and 10% (w/v) phenylenediamine). Samples were observed under the Axiophot Zeiss fluorescence microscope (Germany) with a 460 nm filter for DAPI and a 520 nm filter for FITC-conjugated antibodies, and digital pictures were taken.

**Table 1:** Medium used for cases and controls

**Treatment/control cases**	**10% AF case**	**10% AF control**	**20% AF case**	**20% AF control**	**30% AF case**	**30% AF control**	**10% FBS case**	**10% FBS control**
**Supplemented medium for first 24 hours**	10% AF	10% AF	20% AF	20% AF	30% AF	30% AF	10% FBS	10% FBS
**Supplemented medium for subsequent hours**	10% AF	DMEM/F12 without AF	20% AF	DMEM/F12 without AF	30% AF	DMEM/F12 without AF	10% FBS	DMEM/F12 without FBS

Medium used for AF and FBS treatment and their controls is mentioned in the table above. This condition was continued for immunocytochemistry, ELISA immunoassay and RNA extraction. Each experiment has been explained in the methods.

### RPE cell proliferation and cell death ELISA assays

To quantify the effect of AF on RPE cell proliferation and death, RPE cells from early and mid passages (passages 25 for the data shown) were prepared and analyzed with the cell proliferation and cell death ELISA kits: BrdU colorimetric Cell Proliferation ELISA and Cell Death Detection ELISA kits, (Roche, Germany) according to the manufacturers instructions. Briefly, a 96-well microplate (Nunc.) with 110^4^ cells in each well was prepared with 200 l samples of medium containing 10% AF, 20% AF, 30% AF and 10% FBS. After 24 h incubation at 37C, the medium of each well was changed to fresh medium, control cultures received DMEM/F12 instead of AF or FBS-supplemented medium (Table 1). Cell proliferation (BrdU incorporation immunoassay during DNA synthesis) and cell death (sandwich-enzyme-immunoassay quantification of histone-bound DNA fragments) was assessed using a scanning multi-well spectrophotometer (Titertek multiscan ELISA reader, Labsystems Multiscan, Roden, Netherlands). The calculated proliferation and death rates were compared to the control FBS, DMEM/F12 and positive control (a DNA-histone complex) samples.

### RNA Extraction

RPE cells from mid passages (passage 5 for Real-Time PCR) were trypsinized and then cultured in 75 cm^2^ flasks (Nunc.) at a density of 110^6^ cells per flask, each of which were previously coated with FBS or AF for at least 1 h at 37C. Twenty-four hours after culturing, the medium was exchanged with medium containing 10% FBS, 10% AF, 20% AF, 30% AF or DMEM/F12 as a control and incubated for a further 24 h (Table 1). RPE cells were then trypsinized and precipitated for 5 min at 300*g* and RNA extracted using the RNeasy Plus Mini Kit (Qiagen, Germany) in accordance with the manufacturer's instructions. Total RNA was purified using gDNA eliminator mini spin columns and stored at 70C.

### Real-Time RT-PCR

Primers for *PAX6CHX10VSX-1* and *GAPDH*. (*GAPDH*: house keeping gene used as an internal control) were optimized for the SYBR green assay using Beacon Designer software 7.0 (http://www.primebiosoft.com). Amplicon length and sense and anti-sense primers are presented in Table 2. A cDNA pool was established using superscript III reverse transcriptase (200 U/l) (Invitrogen, Germany) and oligo-dT primers (Fermentas, Belgium) and was subsequently amplified using the iQ SYBR Green supermix kit (Roche, Germany) and the MyiQ apparatus (Bio-Rad, USA). Each reaction (20 l volume) contained 5 l of cDNA, 0.3 l of fast start *Taq*. enzyme, 1 l of forward primer and 1 l of reverse primer. A pre-amplification denaturation was performed at 95C for 8 min, followed by real-time PCR with a thermal profile that included 45 cycles of denaturation at 95C for 30 s, annealing at 57C and 60C for 50 s for *PAX6* and *CHX10*/*VSX-1*, respectively, and then extension at 72C for 50 s. Experiments were performed in triplicate (at least) for each sample. Appropriate serial dilutions were made for each sample, and a standard curve was designed estimating amplification efficiencies. Relative gene expression was calculated using Bio-Rad software (RelQuant UpDate- for relative quantification) according to the 2^-Ct^ method based on the threshold cycle (Ct) values [40].

**Table 2:** Amplicon length and sequences of sense and anti-sense primers

Sequence definition	Product length	Sense primer	Anti-sense primer
*VSX-1*	103	AGACTCCGTGCTCAACTC	TCCTGGCTTCCTTATCATCC
*CHX10*	135	TCGTGATATGCTGCTTGTG	CTGTGGCTTCGTAGATGTC
*PAX6*	120	TTGCTGGAGGATGATGAC	CTATGCTGATTGGTGATGG
*GAPDH*	77	ACAGTCAGCCGCATCTTC	CTCCGACCTTCACCTTCC

Primers for *PAX6*, *CHX10*, *VSX-1* and *GAPDH* were optimized for the SYBR green assay using Beacon Designer software 7.0 (http://www.primebiosoft.com).

## Abbreviations

RPE = Retinal pigment epithelium; AF = Amniotic fluid; AMD = Age related macular degeneration; Ct = Threshold cycle; EMT = Epithelial mescenchymal transition.

## Competing interests

The authors declare that they have no competing interests.

## Authors' contributions

FSJ performed the cell cultures, RNA extraction, cDNA synthesis and real-time PCR, ELISA immunoassay and immunocytochemistry, and drafted the manuscript. HA and ZSS conceived the study and participated in its design and coordination, administratively supported the study, provided study material and finally approved the manuscript. AD and SS performed data analysis and interpretation. MRK and JP provided human samples. MD, SG and AB participated in collection and assembly of data. All authors have read and approved the final manuscript.

## **Authors' information**

**FSJ**: National Institute of Genetic Engineering and Biotechnology, P.O.Box: 14965/161, Pajoohesh Boulevard, 17^th^ Kilometer, Tehran-Karaj Highway, Tehran-Iran, Phone: 9821 44580379, Fax: 9821 44580399, fsanie@yahoo.com. **HA:** Ophthalmic Research Center, no23 Paidar Fard St., Boostan 9 St., Pasdaran Ave., Tehran, 16666, Iran, Phone: 9821 22591616, Fax: 9821 22590607, hahmadieh@gmail.com, ahmadieh@sbmu.ac.ir URL: http://www.orcir.org. **ZSS**: National Institute of Genetic Engineering and Biotechnology, P.O.Box: 14965/161, Pajoohesh Boulevard, 17^th^ Kilometer, Tehran-Karaj Highway, Tehran-Iran, Phone: 9821 44580379, Fax: 9821 44580399, soheili@nigeb.ac.ir. **MD**: National Institute of Genetic Engineering and Biotechnology, P.O.Box: 14965/161, Pajoohesh Boulevard, 17^th^ Kilometer, Tehran-Karaj Highway, Tehran-Iran, Phone: 9821 44580379, Fax: 9821 44580399, davarimlh@gmail.com. **SG**: National Institute of Genetic Engineering and Biotechnology, P.O.Box: 14965/161, Pajoohesh Boulevard, 17^th^ Kilometer, Tehran-Karaj Highway, Tehran-Iran, Phone: 9821 44580379, Fax: 9821 44580399, Shima.ghaderi@gmail.com. **MRK**: Ophthalmic Research Center, no23 Paidar Fard St., Boostan 9 St., Pasdaran Ave., Tehran, 16666, Iran, Phone: 9821 22591616, Fax: 9821 22590607, mrezaie47@orcir.org. **SS**: Iranian Blood Transfusion Organization Research Center, Tehran, Iran, phone: 9821 82052206, Fax: 9821 88601545, shsamie@ibto.ir. **AD**: National Institute of Genetic Engineering and Biotechnology, P.O.Box: 14965/161, Pajoohesh Boulevard, 17^th^ Kilometer, Tehran-Karaj Highway, Tehran-Iran, Phone: 9821 44580377, Fax: 9821 44580399, deezagi@nigeb.ac.ir. **JP**: Department of Obstetrics and Gynecology, Aban General Hospital, Tehran, Iran, Phone: 9821 44580379, Fax: 9821 44580399, jalil@gmail.com. **AB**: National Institute of Genetic Engineering and Biotechnology, P.O.Box: 14965/161, Pajoohesh Boulevard, 17^th^ Kilometer, Tehran-Karaj Highway, Tehran-Iran, Phone: 9821 44580379, Fax: 9821 44580399, bughery@yahoo.com.
